# How to Demonstrate the Presence of Carbonic Oxide in the Air of a Room

**Published:** 1877-09

**Authors:** 


How to Demonstrate the Presence of Carbonic Oxide in the Air of a Room.—According to Prof. Bottcher, a solution of chloride of palladium is a good reagent for carbonic oxide and carburetted hydrogen. Strips of linen or cotton soaked in a concentrated solution, as free from acid as possible, of this substance, assume an intensely black color when brought into contact with the above-named gases.—*Ibid.*

